# Shrunken pore syndrome in relation to morbidity and mortality in the population-based Malmö Diet and Cancer cohort: a generalized propensity score approach

**DOI:** 10.3389/fepid.2025.1661167

**Published:** 2025-09-30

**Authors:** Anna Åkesson, Liana Xhakollari, Agnė Laučytė-Cibulskiene, Anders Grubb, Anders Larsson, Amra Jujic, Martin Magnusson, Anders Christensson, Jonas Björk

**Affiliations:** ^1^ Clinical Studies Sweden - Forum South, Skåne University Hospital, Lund, Sweden; ^2^ Department of Laboratory Medicine, Lund University, Lund, Sweden; ^3^ Department of Clinical Sciences Malmö, Lund University, Malmö, Sweden; ^4^ Department of Nephrology, Skåne University Hospital, Malmö, Sweden; ^5^ Department of Clinical Chemistry, Lund University, Lund, Sweden; ^6^ Department of Medical Sciences, Clinical Chemistry, Uppsala University, Uppsala, Sweden; ^7^ Department of Cardiology, Skåne University Hospital, Malmö, Sweden; ^8^ Wallenberg Centre for Molecular Medicine, Lund University, Lund, Sweden; ^9^ Hypertension in Africa Research Team (HART), North-West University, Potchefstroom, South Africa

**Keywords:** population-based cohort, mortality, refined confounding adjustment, creatinine, cystatin C, eGFR

## Abstract

**Purpose:**

Glomerular filtration rate (GFR) is used for evaluating kidney function. Creatinine and cystatin C levels are the two endogenous substances used to estimate GFR (eGFR_CR_ and eGFR_CYS_). The agreement between these two is reflected by the eGFR_CYS_/eGFR_CR_ ratio. An eGFR_CYS_/eGFR_CR_ ratio <0.70 has been strongly associated with mortality and morbidity. An explanation is a selective decrease in the filtration of substances of different masses, and this condition is referred to as “Shrunken pore syndrome” (SPS). We aim to investigate the prevalence of SPS and its association with morbidity and mortality in a well-characterized population-based cohort.

**Methods:**

The study population consisted of 5,061 individuals from the Malmö Diet and Cancer cardiovascular cohort (MDC-CC) with baseline examinations between 1991 and 1994 and a median follow-up of 25.3 years (IQR = 5.7). The eGFR_CYS_/eGFR_CR_ ratio was categorized into four groups and used to estimate a generalized propensity score for SPS to adjust for confounding factors. Individuals were matched to create a quartet (one from each eGFR_CYS_/eGFR_CR_ ratio category) with similar scores. We related the eGFR_CYS_/eGFR_CR_ ratio to all-cause mortality, incident cardiovascular disease, incident kidney disease, and incident diabetes using Cox proportional hazards models with shared frailty.

**Results:**

SPS was detected in 405 individuals (8.0%). The hazard ratio (HR) for all-cause mortality was 1.6 [95% confidence interval (CI) 1.3–2.0] when comparing individuals with SPS to the reference group (eGFR_CYS_/eGFR_CR_ ratio ≥ 1.0). For incident kidney disease, the association seems to stem from a low eGFR_CYS_ rather than the eGFR_CYS_/eGFR_CR_ ratio. For the other two outcomes, robust and statistically significant associations could not be found.

**Conclusion:**

SPS was prevalent among middle-aged, generally healthy, individuals and led to markedly higher mortality during follow-up.

## 1 Introduction

Glomerular filtration rate (GFR) is considered the clinically most useful way to evaluate kidney function (1). Creatinine and cystatin C are the two endogenous substances commonly used in clinical practice to estimate GFR (eGFR_CR_ and eGFR_CYS_). The Kidney Disease: Improving Global Outcomes (KDIGO) guidelines recommend to base eGFR on both creatinine and cystatin C in clinical practice for classification of chronic kidney disease (CKD) stage and risk calculation of mortality and morbidity (2).

The agreement between eGFR based on creatinine and cystatin C is reflected by the eGFR_CYS_/eGFR_CR_ ratio. In most patients, eGFR_CR_ and eGFR_CYS_ agree, yielding an eGFR_CYS_/eGFR_CR_ ratio close to 1. However, decreased ratios below 0.70 are sometimes observed even in the absence of non-renal factors influencing eGFR_CYS_ or eGFR_CR_ (3). Such low ratios are strongly associated with premature death and morbidity (4–14) and seem robust to the choice of GFR equations (5, 6). These observations have been suggested to be due to a selective decrease in the capacity of the glomerular filtration barrier to filter middle-sized (5–40 kDa) substances, such as cystatin C and beta-2-microglobulin, with virtually intact capacity to filter low molecular mass (<1 kDa) substances, such as water and creatinine, first noted in the third trimester of pregnancy (12, 15, 16). The corresponding condition is hence referred to as “Shrunken pore syndrome (SPS)” (3, 5, 6). Although the designation of the syndrome is based on the theoretical pore model for glomerular filtration, recent structural studies suggest that a thickening of the glomerular basal membrane also can produce a selective decrease in the filtration of middle-sized molecules (12, 16, 17). Whether this is due to shrinking or extension of the pores or to some other mechanism remains to be established.

Since the first description of SPS in 2015 (3), several studies in different patient settings have established SPS as a health condition associated with mortality and morbidity, with prevalence estimates varying from 0.2% to 36% (4, 7, 12, 13, 16, 18, 19). An association to mortality or morbidity in patients with an eGFR_CYS_ value significantly lower than their eGFR_CR_ value has also been found when the difference was used rather than the ratio (20–23).

When SPS has been investigated in general population studies, similar associations with mortality and morbidity have been observed (24, 25). In this study, we aim to further investigate these associations using a well-characterized population-based cohort with register linkage to national registers, allowing for a more refined confounding adjustment.

## 2 Materials and methods

### 2.1 Study cohort

The main study cohort used originates from the Malmö Diet and Cancer (MDC) study, a population-based, prospective study conducted in Malmö in southern Sweden (*n* = 28,449). In MDC, 6,103 subjects were additionally part of a study on the epidemiology of carotid artery disease. This subsample is referred to as the MDC cardiovascular cohort (MDC-CC), with examinations carried out between 1991 and 1994 (26). Samples of cystatin C and creatinine were available for 5,061 individuals, who make up our study cohort. This study was conducted in accordance with the Declaration of Helsinki and was approved by the Ethics Review Board in Lund, Sweden (DNR 2017/846). The manuscript was prepared according to the Strengthening the Reporting of Observational Studies in Epidemiology (STROBE) guidelines (27).

### 2.2 Baseline examinations

Baseline examination included blood sample donations, anthropometric measurements, dietary assessment, and a self-administered questionnaire about alcohol consumption, smoking, physical activity, education, employment status, and medical history.

Hypertension was defined as a history of hypertension, use of antihypertensive medication, or a systolic or diastolic blood pressure ≥140/90 mmHg. Diabetes at baseline was defined as a previous diagnosis of diabetes, use of anti-diabetic medications, or a fasting blood glucose level >6 mmol/L. Current cigarette smoking was defined as use of cigarette within the past year (28). Additional blood sampling, including measurements of cystatin C and creatinine, was carried out in the MDC-CC (28).

### 2.3 Measurements of cystatin C and creatinine in the MDC-CC and the reference cohort

Plasma creatinine (μmol/L) was analyzed at the Department of Clinical Chemistry, Malmö University Hospital, using the Jaffé method, traceable to isotope-dilution mass spectrometry (IDMS). Cystatin C levels (mg/L) were analyzed using a particle-enhanced immunonephelometric assay (N Latex Cystatin; Dade Behring, Deerfield, IL, USA) (21). The cystatin C values in the MDC-CC cohort were analyzed before the introduction of the world calibrator in 2010 (29) and were not standardized according to this (30). The reference value for the method was 0.53–0.95 mg/L.

Non-standardized cystatin C values in the MDC-CC tended to be lower than those in cohorts analyzed after the introduction of the world calibrator (ERM-DA 471/IFCC). We therefore used the association between cystatin C and creatinine levels in a reference cohort to correct the cystatin C values in the MDC-CC (see Statistical analysis). The reference cohort was obtained from non-nephrology units in Uppsala, Sweden, in 2005–2015 and consisted of anonymous data on age, sex, and plasma creatinine and cystatin C levels for patients aged 44–74 years old (*n* = 142,978). All creatinine samples for the reference cohort were analyzed in clinical routine at the hospital with standardized assays traceable to IDMS, and cystatin C samples were determined by an automated particle-enhanced immunoturbidimetric assay and calibrated against the ERM-DA 471/IFCC.

We used the cystatin C-based Caucasian Asian Pediatric Adult (CAPA)-equation (31) to estimate GFR based on corrected cystatin C (eGFR_CYS_) and the Lund-Malmö revised (LMR) equation (32) to estimate GFR based on creatinine (eGFR_CR_). In a Swedish population, CAPA and LMR can advantageously be used, as these equations were developed in Swedish cohorts (31). We categorized eGFR_CYS_/eGFR_CR_ ratio into four groups, with the lowest quartile indicating SPS, to accommodate more of a gradient in the effect.

### 2.4 Endpoints

The endpoints investigated were all-cause mortality, incident cardiovascular disease (CVD) (ICD10 I21–I23, I25; ICD9 410), incident diabetes (ICD10 E100–E149; ICD9 250–259), and incident kidney disease (ICD10 N02–N08, N11–N16, N18–N19; ICD9 585–589). Information on endpoints in MDC was linked with the national patient and cause of death registers kept by the Swedish National Board of Health and Welfare using the personal identity number for each study participant. All participants in the MDC-CC were followed from the baseline examination until the specific endpoint or death or emigration from Sweden or until the end of follow-up (31 December 2018), with a median follow-up of 25.3 years (IQR = 5.7).

### 2.5 Statistical analysis

All statistical analyses were conducted using STATA SE version 16.1 (33) and R v.4.1.0 (34).

#### 2.5.1 Corrected cystatin C using linear regression

For the reference cohort, we restricted the analysis to subjects with cystatin C within the 2.5–97.5 percentile and creatinine within the same range (min–max) as the range in the MDC-CC. Furthermore, the analysis in the reference cohort was weighted to follow the distribution (in deciles) of creatinine as in the MDC-CC. In both cohorts, sex-specific linear regression models were estimated as follows:

$$\ln(\mathrm{cystatin}\;C)=\beta_{0}+\beta_{1}\mathrm{age}+\beta_{2}ln(\mathrm{creatinine})$$

where ln is the natural logarithm and *β*_0_–β_2_ are the estimated regression coefficients. Predicted values from the models were obtained, and corrected cystatin C values for females were calculated as follows:

$$\begin{array}{rl} \ln(\mathrm{cystatin}\;C_{\mathrm{corrected}}) & =\ln(\mathrm{cystatin}\;C_{\mathrm{MDC}\text{-}\mathrm{CC}})\\ & +(\ln({\mathrm{cystatin}\;C}_{\mathrm{reference}\;\mathrm{cohort}}^{\mathrm{predicted}})\\ & -\ln({\mathrm{cystatin}\;C}_{\mathrm{MDC}\text{-}\mathrm{CC}}^{\mathrm{predicted}}))\\ & =\ln(\mathrm{cystatin}\;C_{\mathrm{MDC}\text{-}\mathrm{CC}})\\ & +(\left(-3.805+0.009\mathrm{age}+0.789\ln\;(\mathrm{creatinin}e_{\mathrm{reference}\;\mathrm{cohort}})\right)-\\ & (-2.329+0.009\mathrm{age}\\ & +0.345\ln\;(\mathrm{creatinin}e_{\mathrm{MDC}\text{-}\mathrm{CC}}))) \end{array}$$

and for males:

$$\begin{array}{r} \ln(\mathrm{cystatin}\;C_{\mathrm{corrected}})=\ln(\mathrm{cystatin}\;C_{\mathrm{MDC}\text{-}\mathrm{CC}})\\ +(\ln({\mathrm{cystatin}\;C}_{\mathrm{reference}\;\mathrm{cohort}}^{\mathrm{predicted}})-\ln({\mathrm{cystatin}\;C}_{\mathrm{MDC}\text{-}\mathrm{CC}}^{\mathrm{predicted}}))\\ =\ln(\mathrm{cystatin}\;C_{\mathrm{MDC}\text{-}\mathrm{CC}})\\ +(\left(-3.781+0.006\mathrm{age}+0.787\ln\;(\mathrm{creatinin}e_{\mathrm{reference}\;\mathrm{cohort}})\right)\\ -(-2.445+0.007\mathrm{age}+0.398\ln\;(\mathrm{creatinin}e_{\mathrm{MDC}\text{-}\mathrm{CC}})) \end{array})$$

The values were exponentiated before further analysis.

#### 2.5.2 Generalized propensity score

Propensity score analysis is a method for detailed confounding adjustment in observational studies, by creating matched cohorts of individuals who are balanced with respect to measured confounders (35). The generalized propensity score (GPS) is an extension of the propensity score beyond two groups and is instead calculated as the level of treatment conditional on confounders (36). Here, the treatment was categorized in eGFR_CYS_/eGFR_CR_ ratio intervals, and we estimated the GPS using multinomial logistic regression including all the variables listed in Table 1 as covariates. Then, the predicted probabilities (i.e., the GPS) from the first three categories were used. Next, a greedy matching approach was employed to create quartets of matched individuals. A greedy approach means that the nearest match is found, and then the process repeats itself. The matching generates a quartet consisting of four individuals (one from each eGFR_CYS_/eGFR_CR_ ratio category) with similar scores. To ensure as many quartets as possible, and since each matched quartet is gradually built, i.e., first a pair is found, then a trio, and finally a quartet is formed, matches of higher quality were given priority. Where higher quality was defined as smaller overall distances. A group's (pair, trio, quartet) distance is equal to the sum of all three-dimensional point' individual distances to the Euclidean center. Next, we defined a caliper (the maximum allowed distance) analogous to Rassen et al. (37) as follows:

$$k\times \frac{1}{2}\times 0.2\times \sqrt{\frac{1}{k}\times \sum_{i=1}^{k}\tau_{i}^{2}}$$

where *k* is the number of individual distances, 1/2 since we look at the distance to the center, $\sqrt{1/k\times \sum_{i=1}^{k}\tau_{i}^{2}}$ is the mean variance of the logit of the predicted probabilities, and $0.2\times \sqrt{1/k\times \sum_{i=1}^{k}\tau_{i}^{2}}$ corresponds to 0.2 times the standard deviation of the logit of the propensity score with two treatment groups.

**Table 1:** Baseline characteristics stratified by the ratio of estimated GFR according to eGFR_CYS corr_ and eGFR_CR_ in the Malmö Diet and Cancer cardiovascular cohort and the GPS-matched subset. The lowest category (<0.70) indicates SPS.

Variable	GPS-matched					MCD-CC				
	eGFR_CYS corr_/eGFR_CR_ ratio					eGFR_CYS corr_/eGFR_CR_ ratio				
	Total	<0.70	0.70–0.84	0.85–0.99	≥1.00	Total	<0.70	0.70–0.84	0.85–0.99	≥1.00
*n*	1,332	333	333	333	333	5,061	405	1,377	1,903	1,376
Age, years	59 (48–67)	60 (48–67)	59 (48–67)	58 (47–67)	59 (48–67)	58 (47–67)	60 (48–67)	59 (48–67)	58 (47–67)	59 (47–67)
Missing	0	0	0	0	0	0	0	0	0	0
Female, % (*n*)	59.2 (788)	59.2 (197)	60.7 (202)	56.5 (188)	60.4 (201)	59.1 (2,992)	58.5 (237)	52.4 (722)	60.1 (1,143)	64.7 (890)
Missing	0	0	0	0	0	0	0	0	0	0
Education, % (*n*)										
Primary	53.1 (707)	53.8 (179)	55.3 (184)	49.2 (164)	54.1 (180)	45.6 (2,234)	54.7 (209)	49.5 (655)	45.2 (836)	39.8 (534)
Secondary	29.7 (396)	29.7 (99)	29.1 (97)	30.3 (101)	29.7 (99)	35.1 (1,715)	29.3 (112)	34.7 (460)	35.4 (654)	36.5 (489)
Tertiary	17.2 (229)	16.5 (55)	15.6 (52)	20.4 (68)	16.2 (54)	19.3 (946)	16.0 (61)	15.8 (209)	19.4 (358)	23.7 (318)
Missing	0	0	0	0	0	166	23	53	55	35
Employment status, % (*n*)										
Employed	59.1 (786)	55.1 (183)	59.2 (197)	60.1 (199)	62.2 (207)	65.1 (3,208)	52.1 (201)	61.7 (821)	66.2 (1,230)	70.6 (956)
Retired	32.7 (434)	35.2 (117)	33.3 (111)	29.3 (97)	32.7 (109)	27.7 (1,366)	38.6 (149)	31.7 (422)	25.8 (480)	23.3 (315)
Unemployed	5.3 (71)	7.2 (24)	4.8 (16)	6.9 (23)	2.4 (8)	4.3 (213)	6.7 (26)	3.8 (51)	4.8 (90)	3.4 (46)
Housewife/student	2.9 (38)	2.4 (8)	2.7 (9)	3.6 (12)	2.7 (9)	2.9 (141)	2.6 (10)	2.7 (36)	3.1 (58)	2.7 (37)
Missing	0	0	0	0	0	133	19	47	45	22
Current or latest job, % (*n*)										
Manual worker	45.3 (603)	44.4 (148)	48.3 (161)	42.3 (141)	45.9 (153)	38.6 (1,896)	46.2 (178)	43.6 (579)	36.8 (680)	34.1 (459)
Non-manual worker	43.2 (575)	44.1 (147)	41.4 (138)	43.2 (144)	43.8 (146)	51.3 (2,517)	41.3 (159)	46.4 (616)	53.4 (986)	56.1 (756)
Employer	11.6 (154)	11.4 (38)	10.2 (34)	14.4 (48)	10.2 (34)	10.0 (493)	12.5 (48)	9.9 (132)	9.8 (181)	9.8 (132)
Missing	0	0	0	0	0	155	20	50	56	29
Living alone. % (*n*)	27.4 (365)	27.3 (91)	27.0 (90)	29.1 (97)	26.1 (87)	22.6 (1,145)	22.7 (112)	24.1 (332)	22.0 (419)	20.5 (282)
Missing	0	0	0	0	0	128	18	44	44	22
Smoking habits, % (*n*)										
Current smoker	38.8 (517)	38.7 (129)	38.7 (129)	39.0 (130)	38.7 (129)	22.0 (1,088)	40.8 (159)	28.1 (378)	19.0 (354)	14.5 (197)
Ex-smoker/occasional smoker	29.1 (388)	29.4 (98)	30.3 (101)	29.1 (97)	27.6 (92)	38.0 (1,884)	27.7 (108)	38.2 (513)	38.8 (723)	39.8 (540)
Never smoker	32.1 (427)	31.8 (106)	30.9 (103)	31.8 (106)	33.6 (112)	40.0 (1,980)	31.5 (123)	33.7 (452)	42.2 (786)	45.7 (619)
Missing	0	0	0	0	0	109	15	34	40	20
Physical activity score	6,805 (507–24,414)	6,810 (0–26,236)	6,990 (268–26,355)	6,270 (960–23,458)	7,080 (723–20,985)	6,920 (515–23,900)	6,510 (0–25,547)	6,660 (13–24,645)	6,870 (686–23,490)	7,345 (867–23,743)
Missing	0	0	0	0	0	162	24	52	56	30
BMI, kg/m^2^	26 (20–35)	26 (19–36)	26 (20–36)	26 (19–34)	26 (20–36)	25 (19–35)	26 (19–38)	26 (20–37)	25 (19–34)	24 (20–33)
Missing	0	0	0	0	0	5	0	2	3	0
Blood pressure-lowering drugs, % (*n*)	23.4 (312)	24.6 (82)	24.3 (81)	22.8 (76)	21.9 (73)	16.4 (832)	26.4 (107)	18.6 (256)	14.8 (281)	13.7 (188)
Missing	0	0	0	0	0	0	0	0	0	0
Hypertension, % (*n*)	45.0 (599)	42.3 (154)	45.1 (150)	43.8 (146)	44.7 (149)	38.4 (1,943)	47.9 (194)	41.7 (574)	36.7 (698)	34.7 (477)
Missing	0	0	0	0	0	0	0	0	0	0
Cancer, % (*n*)	7.5 (100)	7.5 (25)	8.4 (28)	6.6 (22)	7.5 (25)	7.9 (400)	8.9 (36)	8.6 (118)	8.3 (158)	6.4 (88)
Missing	0	0	0	0	0	0	0	0	0	0
CVD, % (*n*)	8.6 (115)	9.0 (30)	9.0 (30)	8.4 (28)	8.1 (27)	5.7 (290)	11.9 (48)	7.2 (99)	4.8 (91)	3.8 (52)
Missing	0	0	0	0	0	0	0	0	0	0
Diabetes, % (*n*)	9.8 (131)	10.2 (34)	8.4 (28)	10.5 (35)	10.2 (34)	9.2 (467)	12.8 (52)	11.0 (152)	8.5 (162)	7.3 (101)
Missing	0	0	0	0	0	0	0	0	0	0
CKD, % (*n*)	0.2 (2)	0.3 (1)	0.0 (0)	0.0 (0)	0.3 (1)	0.04 (2)	0.3 (1)	0.0 (0)	0.0 (0)	0.1 (1)
Missing	0	0	0	0	0	0	0	0	0	0

Unless otherwise noted, stated data presented as median (2.5–97.5 percentiles). CKD was not included in the propensity score due to insufficient numbers. GFR, glomerular filtration rate; eGFR, estimated GFR; eGFR_CYS corr_, cystatin C-based estimation of glomerular filtration rate; eGFR_CR_, creatinine-based estimation of glomerular filtration rate; GPS, generalized propensity score; SPS, shrunken pore syndrome; BMI, body mass index; CVD, cardiovascular disease; MCD-CC, Malmö Diet and Cancer cardiovascular cohort; CKD, chronic kidney disease.

Once the matched subset was found, it was essential to assess the similarity of baseline covariates (38). In this case, we calculated standardized mean differences (SMD) for all pair-wise combinations of the quartets, and in a conservative approach, the largest difference was selected. We aimed for a good match across all four groups, and a single large SMD would indicate an overall poorer match.

#### 2.5.3 Cox regression

In the matched analysis, following the recommendations by Austin (38), we account for within-quartet correlation using Cox proportional hazard models with shared frailty to relate the eGFR_CYS_/eGFR_CR_ ratio to the four different endpoints. An unmatched analysis using Cox proportional hazard models was employed, adjusting for all the covariates included in the GPS (see Table 1). As a sensitivity analysis, further adjustment for eGFR_CYS_ was made to investigate whether the associations were entirely driven by the cystatin C level. The results were presented as hazard ratios (HR) with 95% confidence intervals (CI). Prevalent cases were excluded in analyses of incident outcomes.

## 3 Results

Baseline characteristics are presented in Table 1, stratified based on eGFR_CYS_/eGFR_CR_ ratio intervals, for both the GPS-matched subset (*n* = 1,332 individuals in 333 quartets: 26% of the MDC-CC) and the full study cohort (*n* = 5,061). The prevalence of SPS was 8% in the complete study cohort at baseline. Notable imbalances in variables relating to socioeconomic status and smoking habits were observed. In the lowest eGFR_CYS_/eGFR_CR_ ratio group, 46% were manual workers compared with 34% in the highest ratio group. Smoking prevalence in the lowest eGFR_CYS_/eGFR_CR_ ratio group was 41% compared with 15% in the highest group. In contrast, the covariates of the GPS-matched subset were generally balanced. The proportion of current smokers was 39% in both the lowest and highest eGFR_CYS_/eGFR_CR_ ratio group, whereas the proportion of manual workers was 44% and 46%, respectively. The balance achieved across the GFR_CYS_/eGFR_CR_ ratio group can also be assessed with SMD (Supplementary Tables S1, S2). Here too, we see that the imbalance was substantially reduced or eliminated in the matched sample, while large imbalances were found for the individuals that we were not able to match.

Table 2 presents eGFR-related variables. The non-standardized cystatin C levels were lower [0.76 (0.55–1.11), median (2.5–97.5 percentiles)] compared with the corrected values [1.14 (0.80–1.73)] obtained from the correction based on linear regression models. The creatinine and eGFR_CR_ levels were similar across the four groups, whereas cystatin C and eGFR_CYS_ differed considerably.

**Table 2:** Baseline biomarkers of renal function and estimated GFR stratified by the ratio of estimated GFR according to eGFR_CYS corr_ and eGFR_CR_ in the GPS-matched subset from the Malmö Diet and Cancer cardiovascular cohort and in the full Malmö Diet and Cancer cardiovascular cohort. The lowest category (<0.70) indicates SPS.

Variable	GPS-matched					MCD-CC				
	eGFR_CYS corr_/eGFR_CR_ ratio					eGFR_CYS corr_/eGFR_CR_ ratio				
	Total	<0.70	0.70–0.84	0.85–0.99	≥1.00	Total	<0.70	0.70–0.84	0.85–0.99	≥1.00
*n*	1,332	333	333	333	333	5,061	405	1,377	1,903	1,376
Cystatin C, mg/L	0.80 (0.56–1.21)	1.02 (0.86–1.52)	0.85 (0.73–1.03)	0.75 (0.63–0.91)	0.65 (0.49–0.83)	0.76 (0.55–1.11)	1.03 (0.86–1.74)	0.86 (0.73–1.04)	0.75 (0.65–0.89)	0.65 (0.49–0.81)
Cystatin C corrected, mg/L	1.2 (0.82–1.89)	1.51 (1.19–2.44)	1.27 (1.03–1.68)	1.11 (0.88–1.49)	0.98 (0.66–1.40)	1.14 (0.80–1.73)	1.53 (1.19–2.64)	1.27 (1.02–1.67)	1.11 (0.89–1.46)	0.98 (0.71–1.35)
Creatinine, µmol/L^a^	83 (59–118)	83 (56–122)	82 (62–113)	84 (58–111)	84 (58–132)	83 (60–117)	83 (57–139)	83 (61–118)	83 (59–112)	84 (61–125)
eGFR_CYS corr_, ml/min/1.73 m^2^	59 (34–94)	45 (24–61)	56 (39–73)	65 (46–86)	76 (60–122)	63 (38–97)	44 (22–61)	55 (39–74)	65 (47–85)	76 (51–112)
eGFR_CYS_, ml/min/1.73 m^2^	95 (59–143)	72 (44–90)	89 (71–108)	103 (81–126)	120 (91–165)	101 (65–148)	71 (40–90)	88 (70–108)	104 (84–125)	121 (94–168)
eGFR_CR_, ml/min/1.73 m^2^	70 (48–92)	71 (46–94)	70 (51–92)	71 (50–93)	67 (42–90)	70 (49–92)	71 (42–95)	71 (50–92)	71 (51–92)	69 (44–90)
eGFR_CYS corr_/eGFR_CR_ ratio	0.85 (0.54–1.27)	0.65 (0.43–0.69)	0.79 (0.71–0.84)	0.92 (0.85–0.99)	1.09 (1.00–1.56)	0.90 (0.61–1.29)	0.65 (0.41–0.70)	0.79 (0.71–0.85)	0.92 (0.85–0.99)	1.09 (1.00–1.48)
eGFR_CYS_/eGFR_CR_ ratio	1.36 (0.86–2.16)	1.04 (0.78–1.25)	1.26 (1.08–1.52)	1.46 (1.24–1.75)	1.74 (1.48–2.56)	1.44 (0.97–2.16)	1.03 (0.78–1.25)	1.25 (1.05–1.49)	1.46 (1.26–1.73)	1.77 (1.49–2.50)

Data presented as median (2.5–97.5 percentiles). GFR, glomerular filtration rate; eGFR, estimated GFR; eGFR_CYS corr_, cystatin C-based estimation of glomerular filtration rate; eGFR_CR_, creatinine-based estimation of glomerular filtration rate; GPS, generalized propensity score; SPS, shrunken pore syndrome; MCD-CC, Malmö Diet and Cancer cardiovascular cohort.

^a^ Conversion factor: μmol/L/88.4 = mg/dl.

We investigated mortality, incident CVD, incident kidney disease, and incident diabetes for both the GPS-matched subset and the full study cohort (Table 3). In both samples, we observed a higher mortality among those with SPS. The incidence of kidney disease and diabetes was marginally higher for individuals with SPS compared with that of the three other groups, whereas the incidence of CVD was similar across the four groups.

**Table 3:** Incidence rate per 1,000 person-years across the eGFR_CYS corr_/eGFR_CR_ ratio groups in the GPS-matched subset from the Malmö Diet and Cancer cardiovascular cohort and in the full Malmö Diet and Cancer cardiovascular cohort, presented as cases and incidence (95% CI). The lowest category (<0.70) indicates SPS.

Variable	GPS-matched^a^									
	eGFR_CYS corr_/eGFR_CR_ ratio									
	Total		<0.70		0.70–0.84		0.85–0.99		≥1.00	
	Cases	Incidence (95% CI)	Cases	Incidence (95% CI)	Cases	Incidence (95% CI)	Cases	Incidence (95% CI)	Cases	Incidence (95% CI)
Mortality	624	21.7 (20.0–23.4)	175	25.4 (21.8–29.4)	151	21.2 (18.0–24.8)	149	20.1 (17.1–23.6)	149	20.2 (17.1–23.6)
Incident CVD	283	10.6 (9.4–11.9)	71	11.3 (8.9–14.1)	64	9.6 (7.4–12.2)	77	11.3 (9.0–14.1)	71	10.4 (8.2–13.0)
Incident kidney disease	148	5.3 (4.5–6.2)	48	7.3 (5.4–9.6)	34	4.9 (3.4–6.8)	34	4.7 (3.3–6.5)	32	4.5 (3.1–6.2)
Incident Diabetes	201	7.7 (6.7–8.8)	59	9.6 (7.4–12.3)	49	7.6 (5.7–10.0)	56	8.4 (6.4–10.9)	37	5.4 (3.9–7.4)
	MCD-CC^b^									
	eGFR_CYS corr_/eGFR_CR_ ratio									
	Total		<0.70		0.70–0.84		0.85–0.99		≥1.00	
	Cases	Incidence (95% CI)	Cases	Incidence (95% CI)	Cases	Incidence (95% CI)	Cases	Incidence (95% CI)	Cases	Incidence (95% CI)
Mortality	2,017	17.7 (17.0–18.5)	228	28.0 (24.5–31.8)	618	20.7 (19.1–22.4)	715	16.4 (15.2–17.6)	456	14.2 (12.9–15.6)
Incident CVD	996	9.4 (8.8–10.0)	91	12.3 (9.9–15.0)	314	11.4 (10.2–12.7)	364	8.9 (8.0–9.8)	227	7.4 (6.5–8.5)
Incident kidney disease	452	4.1 (3.7–4.5)	65	8.4 (6.5–10.6)	137	4.7 (3.9–5.6)	158	3.7 (3.2–4.3)	92	2.9 (2.4–3.6)
Incident Diabetes	719	6.9 (6.4–7.5)	74	10.3 (8.2–12.9)	210	7.9 (6.9–9.0)	296	7.4 (6.6–8.3)	163	5.4 (4.7–6.3)

GFR, glomerular filtration rate; eGFR, estimated GFR; eGFR_CYS corr_, cystatin C-based estimation of glomerular filtration rate; eGFR_CR_, creatinine-based estimation of glomerular filtration rate; GPS, generalized propensity score; SPS, shrunken pore syndrome; CVD, cardiovascular disease; MCD-CC, Malmö Diet and Cancer cardiovascular cohort; CKD, chronic kidney disease; CI, confidence intervals.

^a^ Prevalent cases of the outcome were excluded before analysis: 55 prevalent CVD, 1 prevalent kidney disease, and 59 prevalent diabetes cases were excluded.

^b^ Prevalent cases of the outcome were excluded before analysis: 130 prevalent CVD, 1 prevalent kidney disease, and 195 prevalent diabetes cases were excluded.

**Figure 1:**
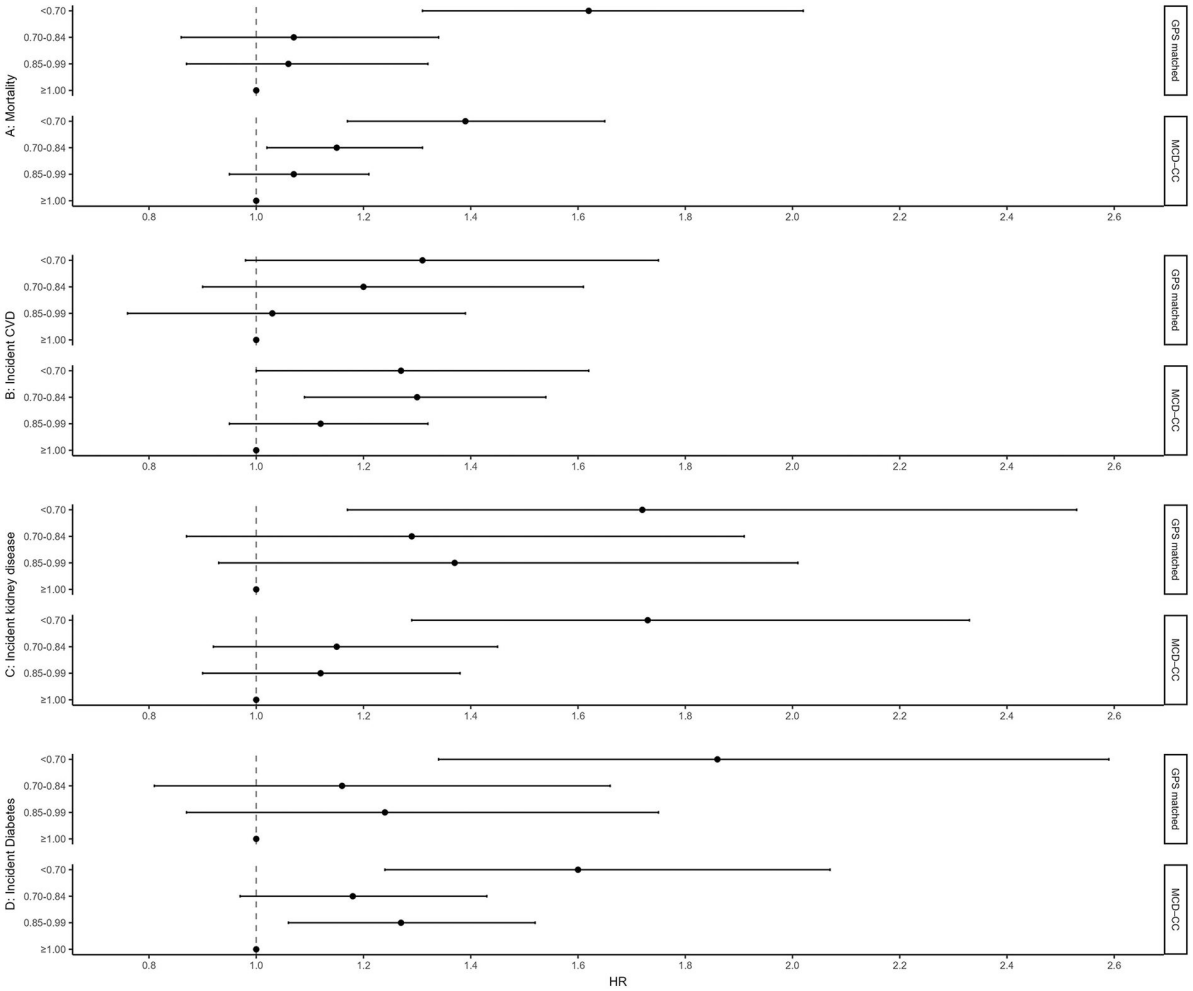
Forest plot of HR and 95% CI for results from Cox regression models relating eGFR_CYS corr_/eGFR_CR_ ratio to all-cause mortality, incident CVD, incident kidney disease, and incident diabetes in the GPS-matched subset from the Malmö Diet and Cancer cardiovascular cohort and in the full Malmö Diet and Cancer cardiovascular cohort. The lowest category (<0.70) indicates SPS. GFR, glomerular filtration rate; eGFR, estimated GFR; eGFR_CYS cor_, cystatin C-based estimation of glomerular filtration rate; eGFR_CR_, creatinine-based estimation of glomerular filtration rate; GPS, generalized propensity score; SPS, shrunken pore syndrome; CVD, cardiovascular disease, MCD-CC, Malmö Diet and Cancer cardiovascular cohort; CKD, chronic kidney disease; HR, hazard ratio; CI, confidence intervals.

Next, we investigated the eGFR_CYS_/eGFR_CR_ ratio for the four endpoints with Cox regression in both samples. In the matched subset, individuals with eGFR_CYS_/eGFR_CR_ ratio <0.70 had higher mortality HR (95% CI), of 1.6 (1.3–2.0) compared with that of individuals with eGFR_CYS_/eGFR_CR_ ratio ≥1.00 (Table 4 and Figure 1). For incident kidney disease, individuals with SPS had 2.1 (1.4–3.4) times higher incidence compared with that of individuals with eGFR_CYS_/eGFR_CR_ ratio ≥1.00. No evident association between the eGFR_CYS_/eGFR_CR_ ratio and incident CVD was found. An association with incident diabetes was suggested. The results in the full study cohort using adjusted Cox regression models (Table 4) mirror those seen for the matched subset, where SPS is significantly associated with three of the endpoints, i.e., mortality [HR 1.5 (1.2–1.8)], incident kidney disease [HR 2.1 (1.5–3.0)] and incident diabetes [HR 1.5 (1.0–2.0)], while for incident CVD, we find no evident association.

**Table 4:** Results from Cox regression models^a^ relating eGFR_CYS corr_/eGFR_CR_ ratio to all-cause mortality, incident CVD, incident kidney disease, and diabetes in the GPS-matched subset from the Malmö Diet and Cancer cardiovascular cohort and in the full Malmö Diet and Cancer cardiovascular cohort. The lowest category (<0.70) indicates SPS.

Variable	All-cause mortality		Incident CVD		Incident kidney disease		Incident diabetes	
	GPS-matched							
	HR (95% CI)	*p*	HR (95% CI)	*p*	HR (95% CI)	*p*	HR (95% CI)	*p*
eGFR_CYS corr_/eGFR_CR_ ratio		<0.001		0.607		0.011		0.066
<0.70	1.6 (1.3–2.0)		1.2 (0.9–1.7)		2.1 (1.4–3.4)		1.6 (1.1–2.2)	
0.70–0.84	1.1 (0.9–1.3)		1.2 (0.8–1.6)		1.5 (0.9–2.4)		1.1 (0.7–1.6)	
0.85–0.99	1.1 (0.9–1.3)		1.0 (0.8–1.4)		1.4 (0.9–2.2)		1.1 (0.7–1.5)	
≥1.00	1.0 (Ref.)		1.0 (Ref.)		1.0 (Ref.)		1.0 (Ref.)	
*n* ^b^	1,332		1,277		1,331		1,273	
	MCD-CC							
	HR (95% CI)	*p*	HR (95% CI)	*p*	HR (95% CI)	*p*	HR (95% CI)	*p*
eGFR_CYS corr_/eGFR_CR_ ratio		0.001		0.214		<0.001		0.038
<0.70	1.4 (1.2–1.7)		1.2 (0.9–1.6)		2.1 (1.5–3.0)		1.4 (1.1–1.9)	
0.70–0.84	1.2 (1.0–1.3)		1.2 (1.0–1.4)		1.2 (0.9–1.6)		1.1 (0.9–1.4)	
0.85–0.99	1.1 (0.9–1.2)		1.1 (0.9–1.3)		1.1 (0.9–1.5)		1.3 (1.0–1.5)	
≥1.00	1.0 (Ref.)		1.0 (Ref.)		1.0 (Ref.)		1.0 (Ref.)	
*n* ^c^	4,819		4,689		4,818		4,638	

GFR, glomerular filtration rate; eGFR, estimated GFR; eGFR_CYS corr_; cystatin C-based estimation of glomerular filtration rate; eGFR_CR_, creatinine-based estimation of glomerular filtration rate; GPS, generalized propensity score; SPS, shrunken pore syndrome; BMI. Body mass index; CVD, cardiovascular disease; MCD-CC, Malmö Diet and Cancer cardiovascular cohort; CKD, chronic kidney disease; HR, hazard ratio; CI, confidence intervals.

^a^ Cox regression with shared frailty in the GPS-matched subset and Cox regression adjusted for female, education, employment status, current or latest job, living alone, smoking habits, physical activity score, BMI, use of blood pressure-lowering drugs, history of hypertension, history of cancer, history of CVD, and history of diabetes.

^b^ Prevalent cases of the outcome were excluded before analysis: 55 prevalent CVD, 1 prevalent kidney disease, and 59 prevalent diabetes cases were excluded.

^c^ Prevalent cases of the outcome were excluded before analysis: 130 prevalent CVD, 1 prevalent kidney disease, and 195 prevalent diabetes cases were excluded.

In the sensitivity analysis, we made further adjustments to the Cox regression models by adding eGFR_CYS_. The association for mortality holds, and individuals with eGFR_CYS_/eGFR_CR_ ratio <0.70 exhibited an increased death rate compared with the reference group [1.4 (1.0–1.9), Table 5]. For incident kidney disease and for incident CVD, we found no evident association. An association with incident diabetes was suggested (Table 5); however, few individuals existed in the reference group for both the eGFR_CYS_/eGFR_CR_ ratio and eGFR_CYS,_ making the results statistically uncertain.

**Table 5:** Results from sensitivity analysis using Cox regression models^a^ relating eGFR_CYS corr_/eGFR_CR_ ratio to all-cause mortality, incident CVD, incident kidney disease, and diabetes in the GPS-matched subset form Malmö Diet and Cancer cardiovascular cohort and in the full Malmö Diet and Cancer cardiovascular cohort adjusting for eGFR_CYS corr_. The lowest category (<0.70) indicates SPS.

Variable	All-cause mortality		Incident CVD		Incident kidney disease		Incident diabetes	
	GPS-matched subset							
	HR (95% CI)	*p*	HR (95% CI)	*p*	HR (95% CI)	*p*	HR (95% CI)	*p*
eGFR_CYS corr_/eGFR_CR_ ratio		0.017		0.968		0.791		0.051
<0.70	1.4 (1.0–1.9)		1.1 (0.7–1.7)		1.3 (0.7–2.4)		2.0 (1.2–3.3)	
0.70–0.84	1.0 (0.8–1.3)		1.1 (0.8–1.6)		1.2 (0.7–2.0)		1.4 (0.9–2.2)	
0.85–0.99	1.0 (0.8–1.3)		1.1 (0.7–1.5)		1.2 (0.8–2.0)		1.3 (0.8–1.9)	
≥1.00	1.0 (Ref.)		1.0 (Ref.)		1.0 (Ref.)		1.0 (Ref.)	
*n* ^b^	1,332		1,277		1,331		1,273	
	MCD-CC							
	HR (95% CI)	*p*	HR (95% CI)	*p*	HR (95% CI)	*p*	HR (95% CI)	*p*
eGFR_CYS corr_/eGFR_CR_ ratio		0.001		0.217		0.437		0.018
<0.70	1.5 (1.2–1.8)		1.3 (0.9–1.7)		1.4 (0.9–2.1)		1.5 (1.0–2.0)	
0.70–0.84	1.3 (1.1–1.5)		1.2 (1.0–1.5)		1.1 (0.8–1.5)		1.1 (0.9–1.5)	
0.85–0.99	1.1 (0.9–1.3)		1.1 (0.9–1.3)		1.0 (0.8–1.3)		1.3 (1.1–1.6)	
≥1.00	1.0 (Ref.)		1.0 (Ref.)		1.0 (Ref.)		1.0 (Ref.)	
*n* ^c^	4,819		4,689		4,818		4,638	

GFR, glomerular filtration rate; eGFR, estimated GFR; eGFR_CYS corr_; cystatin C-based estimation of glomerular filtration rate; eGFR_CR_, creatinine-based estimation of glomerular filtration rate; GPS, generalized propensity score; SPS, shrunken pore syndrome; BMI. Body mass index; CVD, cardiovascular disease; MCD-CC, Malmö Diet and Cancer cardiovascular cohort; CKD, chronic kidney disease; HR, hazard ratio; CI, confidence intervals.

^a^ Cox regression with shared frailty in the GPS-matched subset and Cox regression adjusted for female, education, employment status, current or latest job, living alone, smoking habits, physical activity score, BMI, use of blood pressure-lowering drugs, history of hypertension, history of cancer, history of CVD, and history of diabetes.

^b^ Prevalent cases of the outcome were excluded before analysis: 55 prevalent CVD, 1 prevalent kidney disease, and 59 prevalent diabetes cases were excluded.

^c^ Prevalent cases of the outcome were excluded before analysis: 130 prevalent CVD, 1 prevalent kidney disease, and 195 prevalent diabetes cases were excluded.

Additionally, we also investigated causes of death and how these were distributed across the eGFR_CYS_/eGFR_CR_ ratio groups (Supplementary Table S3). In both the study cohort and the GPS-matched subsample, causes of death were evenly distributed across the eGFR_CYS_/eGFR_CR_ ratio groups.

## 4 Discussion

The prevalence of SPS was 8% in this large population-based cohort of middle-aged individuals. Using a well-characterized population-based cohort with register linkage to national registers, we found that healthy middle-aged individuals with SPS had higher all-cause mortality than individuals without SPS. This association remained after accounting for eGFR_CYS_, suggesting that the effect of SPS on mortality was not driven by GFR or by cystatin C levels *per se*. The association of SPS with a rise in mortality was shown in several studies in different patient cohorts (4, 7–10, 12, 13, 16, 18, 19) and seen in other cohorts of healthy individuals (24, 25). SPS's role in increased mortality is debated with one hypothesis being that the increased mortality in SPS is due to lower kidney function shown by the higher cystatin C levels. In our study, the association between SPS and mortality remained significant in both matched and unmatched groups even after adjustment for eGFR_CYS_, which was confirmed in two other studies (9, 10).

The observed association between SPS and kidney disease was expected since cystatin C is a sensitive marker for kidney function. When adjusting for eGFR_CYS_, the association between SPS and incident kidney disease was no longer apparent, suggesting that this association seems to stem from low eGFR_CYS_ rather than eGFR_CYS_/eGFR_CR_ ratio.

The suggested association of SPS with incident diabetes was unexpected. The individuals with SPS and those without had the same BMI in both matched and unmatched datasets. However, adjustment for eGFR_CYS_ did not affect the association between SPS and diabetes, keeping in mind that due to a limited number of individuals, the results are statistically uncertain and should be interpreted with caution. A reversed association between the eGFR_CYS_/eGFR_CR_ ratio and diabetes has been suggested (12, 17), explained by thickening of the glomerular basal membrane, and would suggest a higher prevalence of SPS among diabetes patients; however, we did not observe this in our study.

A major strength of our study is the use of propensity score matching, a method that attempts to reduce the bias due to confounding variables, meaning that, after matching on the propensity score, we obtain quartets of matched individuals that are similar across all these variables. Thus, our effect estimates in the matched cohort are likely to have lower bias compared with those obtained from just adjusting for confounders in the unmatched cohort. However, a limitation of the propensity score matching is that the number of observations is reduced, since not all individuals find a match, and thus, statistical precision is lowered. Nevertheless, the results seen in the matched subset mirror those seen in the unmatched one with more observations.

Cystatin C was measured before the introduction of the world calibrator in 2010 and was not standardized; however, we used reference material as a way of correcting the cystatin C levels. The cystatin C levels differed in the groups with different eGFR_CYS_/eGFR_CR_ ratio but when adjusted for this difference, SPS was still significantly associated with mortality.

As a limitation of this study, it is worth noting that neither of the two types of analyses presented is shielded from the effects of unmeasured confounding, and thus bias from unmeasured variables cannot be ruled out. One example of unmeasured confounding that may bias the results is that we did not have data on albuminuria levels. Albuminuria is both a strong marker of mortality and an established risk factor for renal and cardiovascular events; thus, the inclusion of albuminuria in the GPS could potentially have influenced the findings of this study.

## 5 Conclusion

Among healthy middle-aged individuals, 8% had SPS in the study cohort. All-cause mortality was markedly higher for individuals with SPS compared with those without SPS. Other studies are needed to investigate the pathophysiology behind SPS and the mechanism behind the association between SPS and mortality.

## Data Availability

The data analyzed in this study are subject to the following licenses/restrictions: The dataset from the Malmö cohorts (39) supporting the conclusions of this article was used under a license and is not available as an open source. Please visit their website for more information. Requests to access these datasets should be directed to https://www.malmo-kohorter.lu.se/malmo-cohorts.
