# Supplementary material for: Shrunken pore syndrome in relation to morbidity and mortality in the population-based Malmö Diet and Cancer cohort: a generalized propensity score approach
Source: Front Epidemiol. 2025 Sep 30;5:1661167. doi: 10.3389/fepid.2025.1661167 (PMC12518306; doi:10.3389/fepid.2025.1661167)
Supplement: Supplementary file 1 [file Datasheet1.docx]

Supplementary material to “Shrunken Pore Syndrome in relation to Morbidity and Mortality in the Population-Based Malmö Diet and Cancer Cohort: A Generalized Propensity Score Approach”

**Supplementary Table S1. Assessing balance across the eGFRCYS corr / eGFRCR ratio–groups using the maximum standardized mean difference form all pairwise comparisons in the GPS matched subset.**

**Supplementary Table S2. Assessing balance across the eGFRCYS corr / eGFRCR ratio–groups using the maximum standardized mean difference form all pairwise comparisons among the individuals that did not find a match.**

**Supplementary Table S3. Total number of deaths and causes of death across the eGFRCYS corr / eGFRCR ratio–groups in Malmö Diet and Cancer Cardiovascular cohort and the GPS matched subset presented as percent (count).**

**Modified STROBE Statement—checklist of items that should be included in reports of observational studies (Cohort/Cross-sectional and case-control studies)**

**Supplementary Table S1. Assessing balance across** **the eGFR_CYS corr_ / eGFR_CR_ ratio–groups using the maximum standardized mean difference form all pairwise comparisons in the GPS matched subset.**

|  | **eGFR_CYS corr_ / eGFR_CR_ ratio** | | | |  |
| --- | --- | --- | --- | --- | --- |
|  | **<0.70** | **0.70–0.84** | **0.85–0.99** | **≥1.00** | **Max SMD** |
| **n** | 333 | 333 | 333 | 333 |  |
| **Female, % (n)** | 59.2 (197) | 60.7 (202) | 56.5 (188) | 60.4 (201) | 0.085 |
| **Smoking habits, % (n)** |  |  |  |  | 0.046 |
| Current smoker | 38.7 (129) | 38.7 (129) | 39.0 (130) | 38.7 (129) |  |
| Ex–smoker /occasional smoker | 29.4 (98) | 30.3 (101) | 29.1 (97) | 27.6 (92) |  |
| Never smoker | 31.8 (106) | 30.9 (103) | 31.8 (106) | 33.6 (112) |  |
| **Education, % (n)** |  |  |  |  | 0.142 |
| Primary | 53.8 (179) | 55.3 (184) | 49.2 (164) | 54.1 (180) |  |
| Secondary | 29.7 (99) | 29.1 (97) | 30.3 (101) | 29.7 (99) |  |
| Tertiary | 16.5 (55) | 15.6 (52) | 20.4 (68) | 16.2 (54) |  |
| **Current or latest job, % (n)** |  |  |  |  | 0.152 |
| Manual worker | 44.4 (148) | 48.3 (161) | 42.3 (141) | 45.9 (153) |  |
| Non–manual worker | 44.1 (147) | 41.4 (138) | 43.2 (144) | 43.8 (146) |  |
| Employer | 11.4 (38) | 10.2 (34) | 14.4 (48) | 10.2 (34) |  |
| **Cancer, % (n)** | 7.5 (25) | 8.4 (28) | 6.6 (22) | 7.5 (25) | 0.068 |
| **CVD, % (n)** | 9.0 (30) | 9.0 (30) | 8.4 (28) | 8.1 (27) | 0.032 |
| **Diabetes, % (n)** | 10.2 (34) | 8.4 (28) | 10.5 (35) | 10.2 (34) | 0.072 |
| **Hypertension, % (n)** | 46.2 (154) | 45.0 (150) | 43.8 (146) | 44.7 (149) | 0.048 |
| **Living alone, % (n)** | 27.3 (91) | 27.0 (90) | 29.1 (97) | 26.1 (87) | 0.067 |
| **Blood pressure lowering drugs, % (n)** | 24.6 (82) | 24.3 (81) | 22.8 (76) | 21.9 (73) | 0.064 |
| **BMI, kg/m^2^** | 26.15 (4.26) | 26.16 (4.19) | 26.15 (3.83) | 26.23 (3.99) | 0.020 |
| **Physical activity score** | 8104.59 (6260.93) | 8317.68 (6343.01) | 7504.07 (5295.06) | 7863.17 (5301.03) | 0.139 |
| **Age** | 58.38 (6.11) | 58.20 (5.77) | 58.12 (5.92) | 58.39 (5.71) | 0.046 |
| **eGFR_CR_. mL/min/1.73 m^2^** | 70.27 (13.36) | 70.77 (10.50) | 71.08 (10.87) | 68.66 (11.94) | 0.212 |
| **eGFR_CYS corr_. mL/min/1.73 m^2^** | 44.44 (9.88) | 55.58 (8.75) | 65.42 (10.23) | 77.08 (17.10) | 2.338 |

Unless otherwise stated data presented as mean (SD). eGFR_CR_ eGFR_CYS corr_; are not included in the propensity score. GFR, glomerular filtration rate; eGFR, estimated GFR; eGFR_CYS corr_; cystatin C-based estimation of glomerular filtration rate; eGFR_CR_, creatinine-based estimation of glomerular filtration rate; GPS, generalized propensity score; CVD, cardiovascular disease; BMI. Body mass index; SMD, standardized mean difference.

**Supplementary Table S2. Assessing balance across the eGFR_CYS corr_ / eGFR_CR_ ratio–groups using the maximum standardized mean difference form all pairwise comparisons among the individuals that did not find a match.**

|  | **eGFR_CYS corr_ / eGFR_CR_ ratio** | | | |  |
| --- | --- | --- | --- | --- | --- |
|  | **<0.70** | **0.70–0.84** | **0.85–0.99** | **≥1.00** | **Max SMD** |
| **n** | 72 | 1044 | 1570 | 1043 |  |
| **Female, % (n)** | 55.6 (40) | 49.8 (520) | 60.8 (955) | 66.1 (689) | 0.334 |
| **Smoking habits, % (n)** |  |  |  |  | 1.183 |
| Current smoker | 52.6 (30) | 24.7 (249) | 14.6 (224) | 6.6 (68) |  |
| Ex–smoker /occasional smoker | 17.5 (10) | 412 (40.8) | 626 (40.9) | 448 (43.8) |  |
| Never smoker | 29.8 (17) | 34.6 (349) | 44.4 (680) | 49.6 (507) |  |
| **Education, % (n)** |  |  |  |  | 0.557 |
| Primary | 61.2 (30) | 47.5 (471) | 44.4 (672) | 35.1 (354) |  |
| Secondary | 26.5 (13) | 36.6 (363) | 36.5 (553) | 38.7 (390) |  |
| Tertiary | 12.2 (6) | 15.8 (157) | 19.1 (290) | 26.2 (264) |  |
| **Current or latest job, % (n)** |  |  |  |  | 0.812 |
| Manual worker | 57.7 (30) | 42.1 (418) | 35.6 (539) | 30.2 (306) |  |
| Non–manual worker | 23.1 (12) | 48.1 (478) | 55.6 (842) | 60.2 (610) |  |
| Employer | 19.2 (10) | 9.9 (98) | 8.8 (133) | 9.7 (98) |  |
| **Cancer, % (n)** | 15.3 (11) | 8.6 (90) | 8.7 (136) | 6.0 (63) | 0.303 |
| **CVD, % (n)** | 25.0 (18) | 6.6 (69) | 4.0 (63) | 2.4 (25) | 0.696 |
| **Diabetes, % (n)** | 25.0 (18) | 11.9 (124) | 8.1 (127) | 6.4 (67) | 0.528 |
| **Hypertension, % (n)** | 55.6 (40) | 40.6 (424) | 35.2 (552) | 31.4 (328) | 0.501 |
| **Living alone, % (n)** | 29.2 (21) | 23.2 (242) | 20.5 (322) | 18.7 (195) | 0.247 |
| **Blood pressure lowering drugs, % (n)** | 34.7 (25) | 16.8 (175) | 13.1 (205) | 11.0 (115) | 0.588 |
| **BMI, kg/m^2^** | 29.90 (5.52) | 26.55 (4.22) | 25.44 (3.72) | 24.42 (3.12) | 1.222 |
| **Physical activity score** | 4923.42 (3693.99) | 7867.96 (5892.23) | 8320.20 (5999.42) | 8604.83 (5763.53) | 0.761 |
| **Age** | 59.60 (6.22) | 57.93 (5.89) | 57.21 (5.88) | 56.63 (5.96) | 0.489 |
| **eGFR_CR_. mL/min/1.73 m^2^** | 68.71 (15.45) | 71.51 (10.82) | 70.89 (10.19) | 69.01 (10.98) | 0.229 |
| **eGFR_CYS corr_ mL/min/1.73 m^2^** | 42.24 (11.20) | 55.88 (8.94) | 65.41 (9.81) | 77.55 (15.03) | 2.664 |

Unless otherwise stated data presented as mean (SD). eGFR_CR_ eGFR_CYS corr_; are not included in the propensity score. GFR, glomerular filtration rate; eGFR, estimated GFR; eGFR_CYS corr_; cystatin C-based estimation of glomerular filtration rate; eGFR_CR_, creatinine-based estimation of glomerular filtration rate; GPS, generalized propensity score; CVD, cardiovascular disease; BMI. Body mass index; SMD, standardized mean difference.

**Supplementary Table S3. Total number of deaths and causes of death across the eGFR_CYS corr_ / eGFR_CR_ ratio–groups in Malmö Diet and Cancer Cardiovascular cohort and the GPS matched subset presented as percent (count).**

|  | **GPS Matched** | | | | | **MCD–CC** | | | | |
| --- | --- | --- | --- | --- | --- | --- | --- | --- | --- | --- |
|  |  | **eGFR_CYS corr_ / eGFR_CR_ ratio** | | | |  | **eGFR_CYS corr_ / eGFR_CR_ ratio** | | | |
|  | **Total** | **<0.70** | **0.70–0.84** | **0.85–0.99** | **≥1.00** | **Total** | **<0.70** | **0.70–0.84** | **0.85–0.99** | **≥1.00** |
| **n** | 1 332 | 333 | 333 | 333 | 333 | 5 061 | 405 | 1 377 | 1 903 | 1 376 |
| **Total number of deaths** | 46.9 (624) | 52.6 (175) | 45.4 (151) | 44.7 (149) | 44.7 (149) | 39.9 (2 017) | 56.3 (228) | 44.9 (618) | 37.6 (715) | 33.1 (456) |
| **Cancer** | 15.6 (210) | 13.8 (46) | 18.6 (62) | 14.4 (48) | 16.2 (54) | 13.9 (705) | 13.3 (54) | 16.9 (233) | 13.0 (248) | 12.4 (170) |
| **CVD** | 14.6 (194) | 16.5 (55) | 14.1 (47) | 13.5 (45) | 14.1 (47) | 12.1 (610) | 20.0 (81) | 13.8 (190) | 10.5 (200) | 10.1 (139) |
| **Diabetes** | 1.0 (13) | 1.2 (4) | 0.9 (3) | 1.2 (4) | 0.6 (2) | 0.9 (44) | 1.0 (4) | 1.2 (16) | 0.6 (12) | 0.9 (12) |
| **Kidney disease** | 0.5 (6) | 0.9 (3) | 0 (0) | 0.6 (2) | 0.3 (1) | 0.4 (18) | 1.2 (5) | 0.4 (5) | 0.3 (6) | 0.2 (2) |
| **Other causes** | 15.1 (201) | 20.1 (67) | 11.7 (39) | 15.0 (50) | 13.5 (45) | 12.7 (640) | 20.7 (84) | 12.6 (174) | 13.1 (249) | 9.7 (133) |

Where other causes largely consist of respiratory, neurological, psychiatric, and infectious diseases. eGFR, estimated GFR; eGFR_CYS corr_, cystatin C-based estimation of glomerular filtration rate; eGFR_CR_, creatinine-based estimation of glomerular filtration rate; GPS, generalized propensity score; CVD, cardiovascular disease, MCD–CC, Malmö Diet and Cancer Cardiovascular cohort.

**Modified STROBE Statement—checklist of items that should be included in reports of observational studies (Cohort/Cross-sectional and case-control studies)**

|  | Item No | Recommendation |
| --- | --- | --- |
| **Title and abstract** | 1 | (*a*) Indicate the study’s design with a commonly used term in the title or the abstract  **Done** |
|  |  | (*b*) Provide in the abstract an informative and balanced summary of what was done and what was found  **Done** |
| Introduction | | |
| Background/rationale | 2 | Explain the scientific background and rationale for the investigation being reported  **Done (page 3)** |
| Objectives | 3 | State specific objectives, including any prespecified hypotheses  **Done (page 3)** |
| Methods | | |
| Study design | 4 | Present key elements of study design early in the paper  **Done (page 4-5)** |
| Setting | 5 | Describe the setting, locations, and relevant dates, including periods of recruitment, exposure, follow-up, and data collection  **Done (pages 4-5)** |
| Participants | 6 | (*a*) *Cohort study*—Give the eligibility criteria, and the sources and methods of selection of participants. Describe methods of follow-up  **Done (page 4-5)**  *Case-control study*—Give the eligibility criteria, and the sources and methods of case ascertainment and control selection. Give the rationale for the choice of cases and controls  *Cross-sectional study*—Give the eligibility criteria, and the sources and methods of selection of participants |
| Variables | 7 | Clearly define all outcomes, exposures, predictors, potential confounders, and effect modifiers. Give diagnostic criteria, if applicable  **Done (page 4-5)** |
| Data sources/ measurement | 8* | For each variable of interest, give sources of data and details of methods of assessment (measurement).  **Done (page 4-6)** |
| Bias | 9 | Describe any efforts to address potential sources of bias  **Done (page 8)** |
| Study size | 10 | Explain how the study size was arrived at (if applicable)  **Not applicable (based on an existing study cohort)** |
| Quantitative variables | 11 | Explain how quantitative variables were handled in the analyses. If applicable, describe which groupings were chosen and why  **Done (e.g. see page 4)** |
| Statistical methods | 12 | (*a*) Describe all statistical methods, including those used to control for confounding  **Done (pages 5-6)** |
|  |  | (*b*) Describe any methods used to examine subgroups and interactions  **Not applicable, no subgroups investigated** |
|  |  | (*c*) Explain how missing data were addressed  **Analysis based on complete case** |
|  |  | (*d*) *Cohort study*—If applicable, explain how loss to follow-up was addressed  **Not applicable (no losses to follow up)**  *Case-control study*—If applicable, explain how matching of cases and controls was addressed  *Cross-sectional study*—If applicable, describe analytical methods taking account of sampling strategy |
|  |  | (*e*) Describe any sensitivity analyses  **Done (page 7)** |
| Results | | |
| Participants | 13* | (a) Report numbers of individuals at each stage of study—eg numbers potentially eligible, examined for eligibility, confirmed eligible, included in the study, completing follow-up, and analyzed  **Done (see page 3-4)** |
|  |  | (c) **Use of a flow diagram**  **Not used** |
| Descriptive data | 14* | (a) Give characteristics of study participants (eg demographic, clinical, social) and information on exposures and potential confounders  **Done (Table 1)** |
|  |  | (b) Indicate number of participants with missing data for each variable of interest  **Done (Table 1)** |
|  |  | (c) *Cohort study*—Summarize follow-up time (eg, average and total amount)  **Done (page 4; median follow up time reported)** |
| Outcome data | 15* | *Cohort study*—Report numbers of outcome events or summary measures over time  **Done (page 6-7; Table 3)** |
|  |  | *Case-control study—*Report numbers in each exposure category, or summary measures of exposure |
|  |  | *Cross-sectional study—*Report numbers of outcome events or summary measures |
| Main results | 16 | (*a*) Give unadjusted estimates and, if applicable, confounder-adjusted estimates and their precision (eg, 95% confidence interval). Make clear which confounders were adjusted for and why they were included  **Done (see. Table 4 and 5)** |
| Other analyses | 17 | Report other analyses done—eg analyses of subgroups and interactions, and sensitivity analyses  **Done (pages 7)** |
| Discussion | | |
| Key results | 18 | Summarize key results with reference to study objectives  **Done (pages 7-8)** |
| Limitations | 19 | Discuss limitations of the study, taking into account sources of potential bias or imprecision.  **Done (page 7-8)**  Discuss both direction and magnitude of any potential bias |
| Interpretation | 20 | Give a cautious overall interpretation of results considering objectives, limitations, multiplicity of analyses, results from similar studies, and other relevant evidence  **Done (page 7-8)** |
| Generalizability | 21 | Discuss the generalizability (external validity) of the study results  **Done (page 7-8).** |

*Give information separately for cases and controls in case-control studies and, if applicable, for exposed and unexposed groups in cohort and cross-sectional studies.

**Note:** An Explanation and Elaboration article discusses each checklist item and gives methodological background and published examples of transparent reporting. The STROBE checklist is best used in conjunction with this article (freely available on the Web sites of PLoS Medicine at http://www.plosmedicine.org/, Annals of Internal Medicine at http://www.annals.org/, and Epidemiology at http://www.epidem.com/). Information on the STROBE Initiative is available at www.strobe-statement.org.
